# Risk Factor Analysis and Prognosis of Dysphagia in Craniocerebral Injury Patients: Implications for Targeted Nursing Strategies

**DOI:** 10.1002/brb3.71347

**Published:** 2026-03-26

**Authors:** Qingye Zhang, Yuxia Pan, Hongyi Sun, Fang Yang

**Affiliations:** ^1^ Intensive Care Unit Taizhou Hospital of Traditional Chinese Medicine (Taizhou Affiliated Hospital of Nanjing University of Chinese Medicine) Taizhou China; ^2^ Department of Nursing Taizhou Hospital of Traditional Chinese Medicine (Taizhou Affiliated Hospital of Nanjing University of Chinese Medicine) Taizhou China

**Keywords:** dysphagia, nursing, prognosis, risk factors

## Abstract

**Background:**

This study aims to develop a predictive model for risk factors associated with dysphagia in patients with craniocerebral injury and to propose targeted nursing and rehabilitation interventions based on this model to improve patient outcomes.

**Methods:**

A retrospective analysis was conducted on clinical data from 150 patients with craniocerebral injury admitted between June 2022 and June 2024. Patients were divided into dysphagia (*n *= 62) and control (*n *= 88) groups based on the presence or absence of dysphagia. Univariate analysis and binary logistic regression were performed to identify independent risk factors for dysphagia, based on which a nomogram prediction model was subsequently constructed.

**Results:**

Binary logistic regression identified vagus nerve injury, tracheotomy/cannulation, mechanical ventilation, and severe aphasia as independent risk factors for dysphagia, while a higher Glasgow Coma Scale (GCS) score served as a protective factor. The nomogram model based on these variables demonstrated good predictive performance, with an AUC of 0.877 (95% CI: 0.823–0.931) as validated by internal bootstrap resampling. Decision curve analysis showed no significant difference between predicted and observed outcomes (*X* ^2^ = 5.6728, *p* = 0.6838), and the absolute error between predicted and actual values was 0.038, indicating strong clinical utility.

**Conclusion:**

Vagus nerve injury, tracheotomy/cannulation, mechanical ventilation, severe aphasia, and GCS score are independent factors influencing the risk of dysphagia in craniocerebral injury patients. The developed predictive model demonstrates high accuracy and may provide a valuable reference for optimizing preventive nursing strategies and reducing the incidence of dysphagia.

## Graphical abstract

This graphical abstract summarizes the identification of independent risk factors for dysphagia in patients with craniocerebral injury and illustrates the development of a nomogram‐based prediction model. The model enables early risk stratification and supports targeted nursing and rehabilitation interventions to reduce dysphagia incidence and improve patient outcomes.

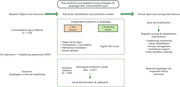

## 1 Introduction

Craniocerebral injury, commonly caused by traffic accidents, falls from height, and blunt force trauma, is a frequent and severe condition in neurosurgical practice. It is characterized by complex pathophysiology and imposes a substantial physical, psychological, and socioeconomic burden on patients, families, and society at large (Ma et al. 2024). During the rehabilitation process, swallowing function plays a critical role in ensuring adequate nutritional intake and protecting the respiratory tract. Dysphagia following craniocerebral injury can lead to feeding difficulties and malnutrition, hindering physical recovery. Moreover, it increases the risk of aspiration and aspiration pneumonia, potentially life‐threatening complications that can prolong hospitalization and escalate healthcare costs (Ni et al. 2022; Cairncross et al. 2022). Although progress has been made in understanding dysphagia in patients with craniocerebral injury, current research remains limited in terms of identifying and comprehensively evaluating its risk factors. Furthermore, effective predictive models for dysphagia risk are lacking (Wang et al. 2022). Developing a robust and accurate risk prediction model would enable clinicians to identify high‐risk patients early, allowing for the implementation of personalized preventive interventions aimed at reducing the incidence of dysphagia and improving clinical outcomes (Beucler and Rambolarimanana 2024).

This study aims to construct a predictive model for identifying risk factors associated with dysphagia in patients with craniocerebral injury and to propose targeted interventions based on the model. By analyzing and predicting relevant risk factors, this study seeks to provide evidence‐based guidance for clinical decision‐making. We also attempt to optimize nursing and rehabilitation strategies, enhance recovery of swallowing function and quality of life, and ultimately reduce the burden on families and society. The findings are expected to have significant clinical and social implications.

## 2 Materials and Methods

### 2.1 Data Collection

Based on the study by Banda et al. (2022), which reported a dysphagia incidence of 42% in patients with craniocerebral injury, PASS software was used for sample size estimation. The logistic regression module was selected with the following parameters: solve for: sample size; hypothesis test type: two‐sided; power: 0.90; alpha (*α*): 0.05; baseline probability (*P*_0_, probability that *Y* = 1): 0.42; alternative probability (*P*_1_, probability that *Y* = 1): set to range from 0.30 to 0.40 in increments of 0.05; R‐squared of *X*_1_ with other *X*’s: 0.0; *X*_1_ (independent variable of interest): continuous (normal distribution); expected dropout rate: 20%. The final estimated sample size was 156 cases. After excluding five cases that did not meet the inclusion or exclusion criteria, 150 patients were ultimately included in the study. This retrospective analysis was conducted on the clinical data of 150 patients (83 males; 67 females; with a mean age of 57.68 ± 4.49 years) with craniocerebral injury admitted between June 2022 and June 2024. Among them, 83 were male, and 67 were female. The average time from injury to hospital admission was 5.82 ± 1.39 h. The mean body mass index (BMI) was 23.76 ± 1.54 kg/m^2^. Causes of injury included crush injury by heavy objects (*n *= 50), falls (*n *= 53), and traffic accidents (*n *= 47). Treatment modalities included emergency surgery (*n *= 91) and elective surgery (*n *= 59). Aphasia severity was classified as mild (*n *= 62), moderate (*n *= 60), or severe (*n *= 28). All eligible patients with craniocerebral injury were initially admitted to the intensive care unit (ICU) between June 2022 and June 2024. After stabilization, some patients were transferred to general wards according to routine clinical practice.

Inclusion criteria were set as: (1) diagnosis of craniocerebral injury confirmed by magnetic resonance imaging (MRI) or cranial computed tomography (CT), with a documented history of craniocerebral trauma; (2) ability to cooperate with the water swallow test (WST); (3) with complete clinical data.

Exclusion criteria: (1) pre‐existing dysphagia prior to the current illness; (2) coagulation disorders or severe electrolyte imbalances; (3) presence of hematologic diseases; (4) complete loss of oropharyngeal motor function; (5) inability to maintain a sitting position even with assistance; (6) craniocerebral injury during pregnancy; (7) history of metal implants in the head or neck; (8) history of cervical spinal cord injury, cervical fracture, or oropharyngeal surgeries.

### 2.2 Diagnostic Criteria

Patients were consecutively enrolled based on predefined eligibility criteria rather than the availability of WST results. The WST is routinely performed in our ICU once patients meet safety and cooperation requirements, and WST results were retrospectively retrieved from medical records. Patients without documented WST assessments or with missing key variables were excluded. Swallowing function was assessed using the WST. Patients were first instructed to drink 1, 3, and 5 mL of water. If no abnormalities were observed, they were then asked to drink 30 mL of water under normal conditions. The presence of dysphagia was determined by monitoring for symptoms such as coughing, altered drinking frequency, and prolonged drinking time. Dysphagia was diagnosed if symptoms such as poor cooperation, weakened palatal movement, diminished pharyngeal reflex, or coughing during drinking were observed (Elizabeth et al. 2022). Based on swallowing function, patients were divided into two groups: normal swallowing function group (*n *= 88) and dysphagia group (*n *= 62). The WST was performed after clinical stabilization. In non‐intubated patients, the initial assessment was typically conducted within 24–48 h after admission. In patients who required invasive mechanical ventilation, WST was performed only after extubation, following a minimum observation period of approximately 3 h, once respiratory stability and adequate alertness were confirmed. Reassessment was performed every 24–48 h or when there were clinically significant changes in neurological or airway status.

### 2.3 Study Variables and Procedures

The following variables were collected and compared between the two groups: sex, age, BMI, time from injury to admission, cause of injury, treatment method, pulmonary infection, serum albumin (ALB), neutrophil count (NE), white blood cell count (WBC), blood lactate level, prothrombin time (PT), procalcitonin (PCT), vagus nerve injury, tracheotomy/cannulation, mechanical ventilation, aphasia severity, and the Glasgow Coma Scale (GCS) score. Aphasia severity was graded based on the Boston Classification (Kim and Yang 2022): (1) mild: fluent speech and relatively intact communication, though with some comprehension difficulties when processing complex information; (2) moderate: simple communication on familiar topics with support, but with inability to express ideas about unfamiliar topics; (3) severe: marked language impairment, requiring frequent guessing or prompting; unable to produce coherent speech and limited understanding of others' language.

For GCS scoring (Bodien et al. 2021): it assesses motor response, verbal response, and eye opening, with a total score of 15. Lower scores indicate more severe consciousness impairment. For blood sampling and analysis: 3 mL of venous blood was collected from the cubital vein of each patient. Serum was separated by centrifugation at 3000 rpm for 5 min. Laboratory parameters, including NE, WBCs, ALB, blood lactate, PT, and PCT, were measured using the Advia 2400 automatic biochemical analyzer. Extended analyses adjusting for illness severity and ventilation duration were conducted as robustness checks and did not materially alter the primary findings.

The GCS score recorded in this study reflects baseline neurological status at admission. The WST was performed only when patients achieved sufficient alertness and cooperation, as determined by routine clinical assessment, including the ability to follow simple commands, maintain sitting postures with assistance, and demonstrate stable airway and respiratory status.

### 2.4 Statistical Analysis

Statistical analyses were performed using SPSS version 27.0 (IBM Corp., Armonk, NY, USA). Continuous variables conforming to normal distribution were expressed as $\bar{x}\pm s$ and compared using the *t*‐test. Categorical variables were expressed as counts and percentages (*n*, %) and compared using the *χ* ^2^ test. A significance level of *α* = 0.05 was adopted. Univariate analysis and binary logistic regression were employed to identify independent risk factors for dysphagia in patients with craniocerebral injury. Significant independent variables were then incorporated into a nomogram prediction model using R software. To minimize model overfitting, internal validation was performed using the Bootstrap resampling method. Model performance was evaluated by the area under the receiver operating characteristic (ROC) curve, and calibration was assessed with calibration plots. To assess potential multicollinearity among predictors included in the multivariable logistic regression model, variance inflation factors (VIFs) were calculated. Sensitivity analyses were performed to evaluate the robustness of the multivariable model. First, patients with extremely low baseline neurological status (baseline GCS ≤ 6) were excluded, and the logistic regression analysis was repeated. In addition, stratified analyses were conducted according to baseline GCS categories (≤ 7, 8–9, and ≥ 10) to examine the consistency of the association between mechanical ventilation and dysphagia across different levels of neurological severity.

## 3 Results

### 3.1 Univariate Analysis

There were no statistically significant differences between the dysphagia group and the normal swallowing function group in terms of sex, cause of injury, treatment type, presence of pulmonary infection, age, BMI, time from injury to hospital admission, ALB, NE, WBC, serum lactate, PT, or PCT levels (*p* > 0.05). However, significant differences were observed between the two groups in terms of vagus nerve injury, tracheotomy/cannulation, mechanical ventilation, severity of aphasia, and GCS score (*p* < 0.05). Details are shown in Table 1.

**TABLE 1:** Comparison of variables between groups.

Variables	Classification	Normal swallowing function group (*n* = 88)	Dysphagia group (*n* = 62)	*χ* ^2^/*t*	*p*
Sex	Male	49 (55.68)	34 (54.84)	0.010	0.919
	Female	39 (44.32)	28 (45.16)		
Cause of injury	Crushed by heavy objects	29 (32.95)	21 (33.87)	0.026	0.987
	Falls	31 (35.23)	22 (35.48)		
	Traffic accidents	28 (31.82)	19 (30.65)		
Vagus nerve injury	Presence	34 (38.64)	39 (62.90)	8.574	0.003
	Absence	54 (61.36)	23 (37.10)		
Tracheotomy/cannulation	Presence	17 (19.32)	26 (41.94)	9.099	0.003
	Absence	71 (80.68)	36 (58.06)		
Mechanical ventilation	Presence	15 (17.05)	24 (38.71)	8.873	0.003
	Absence	73 (82.95)	38 (61.29)		
Aphasia severity	Mild	40 (45.45)	22 (35.48)	6.441	0.040
	Moderate	36 (40.91)	21 (33.87)		
	Severe	12 (13.64)	19 (30.65)		
Treatment method	Emergency surgery	52 (59.09)	39 (62.90)	0.222	0.638
	Elective surgery	36 (40.91)	23 (37.10)		
Pulmonary infection	Presence	23 (26.14)	18 (29.03)	0.154	0.695
	Absence	65 (73.86)	44 (70.97)		
Age (years)		57.49 ± 4.15	57.98 ± 4.62	0.687	0.493
BMI (kg/m^2^)		23.65 ± 1.78	23.82 ± 1.54	0.625	0.533
Time from injury to admission (h)		5.86 ± 1.75	5.79 ± 1.52	0.267	0.790
GCS scoring (score)		9.74 ± 1.46	7.32 ± 1.86	8.902	< 0.001
ALB (g/L)		36.52 ± 4.21	36.97 ± 4.53	0.627	0.532
NE (×10^9^)		3.59 ± 1.32	3.78 ± 1.44	0.839	0.403
WBC (×10^9^)		7.48 ± 2.31	7.59 ± 2.54	0.274	0.784
Blood lactate level (mmol/L)		2.68 ± 0.42	2.71 ± 0.39	0.448	0.655
PT (s)		12.54 ± 2.64	12.87 ± 2.51	0.771	0.438
PCT (mg/L)		1.32 ± 0.19	1.31 ± 0.28	0.285	0.776

### 3.2 Binary Logistic Regression Analysis

Variables with *p* < 0.05 in the univariate analysis were included in the binary logistic regression model, with specific variable assignments detailed in Table 2. Multivariate analysis revealed that vagus nerve injury, tracheotomy/cannulation, mechanical ventilation, and severe aphasia were independent risk factors for dysphagia in patients with craniocerebral injury, while a higher GCS score was identified as a protective factor (*p* < 0.05). See Table 3 for detailed results. To further address potential confounding by overall illness severity and intensity of respiratory support, additional multivariable analyses were performed by incorporating validated severity scores (APACHE‐II or SOFA) and duration of mechanical ventilation. After adjustment for these factors, mechanical ventilation remained independently associated with an increased risk of dysphagia, and baseline GCS continued to show a protective effect. The magnitude and direction of the associations were largely unchanged, supporting the robustness of the primary model.

**TABLE 2:** Variable assignment for logistic regression analysis.

No.	Variables	Value assignment
*Y*	Swallowing function	0 = Normal swallowing function group; 1 = dysphagia group
*X*_1_	Vagus nerve injury	0 = Presence; 1 = absence
*X*_2_	Tracheotomy/cannulation	0 = Presence; 1 = absence
*X*_3_	Mechanical ventilation	0 = Presence; 1 = absence
*X*_4_	Aphasia severity	0 = Mild; 1 = moderate; 2 = severe
*X*_5_	GCS score	Included using actual values

**TABLE 3:** Binary logistic regression analysis of risk factors for dysphagia in patients with craniocerebral injury.

Variables	*B*	S.E.	Wald	*p*	OR	95% CI	
						Lower limit	Upper limit
Vagus nerve injury (1)	0.944	0.455	4.310	0.038	2.569	1.054	6.262
Tracheotomy/cannulation (1)	1.143	0.514	4.941	0.026	3.138	1.145	8.599
Mechanical ventilation (1)	1.234	0.495	6.206	0.013	3.435	1.301	9.071
Aphasia severity	0.656	0.320	4.193	0.041	1.927	1.028	3.609
GCS score	−0.902	0.175	26.679	0.000	0.406	0.288	0.571
Constant	5.103	1.555	10.769	0.001	164.491		

All variables demonstrated low VIF values (vagus nerve injury: 1.045; tracheotomy: 1.087; mechanical ventilation: 1.026; aphasia severity: 1.043; baseline GCS score: 1.110), indicating no evidence of significant multicollinearity.

Collinearity diagnostics showed no significant multicollinearity among the included variables, with all VIFs close to 1. In sensitivity analyses excluding patients with a baseline GCS score ≤ 6 (*n *= 25), the main findings remained unchanged. Mechanical ventilation was still significantly associated with an increased risk of dysphagia (OR = 3.404, *p* = 0.013), whereas baseline GCS remained a protective factor (OR = 0.495, *p* = 0.001). Further stratified analyses according to baseline GCS categories (≤ 7, 8–9, and ≥ 10) showed that the direction of the association between mechanical ventilation and dysphagia was consistent across all strata, with odds ratios greater than 1. Although statistical significance was attenuated after stratification (likely due to reduced sample sizes within each subgroup), these results suggest a stable trend indicating an increased risk of dysphagia associated with mechanical ventilation across different levels of neurological severity (Table S1).

Among the 39 patients who required invasive mechanical ventilation, 15 patients were in the normal swallowing function group, and 24 patients were in the dysphagia group. Patients who developed dysphagia had a longer duration of mechanical ventilation compared with those with preserved swallowing function, with a median duration of 7.5 days (IQR, 5.0–11.0) versus 4.0 days (IQR, 2.5–6.0), respectively. This distribution supports a dose–response relationship between prolonged mechanical ventilation and dysphagia and is consistent with the direction observed in the primary multivariable analysis.

### 3.3 Construction of a Nomogram Prediction Model for Dysphagia Risk

**FIGURE 1:**
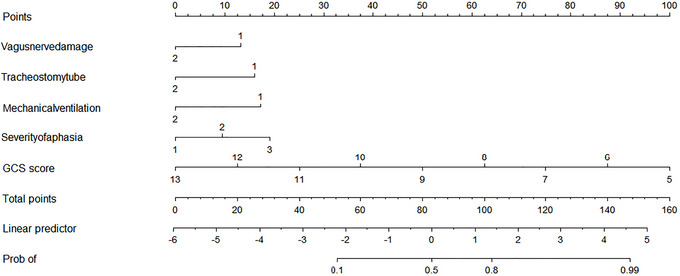
Nomogram for predicting dysphagia risk in patients with craniocerebral injury.

The independent risk factors identified: vagus nerve injury, tracheotomy/cannulation, mechanical ventilation, aphasia severity, and GCS score. These factors were incorporated into the R software to construct a nomogram prediction model. Among these variables, the GCS score contributed the highest weight (see Figure 1).

**FIGURE 2:**
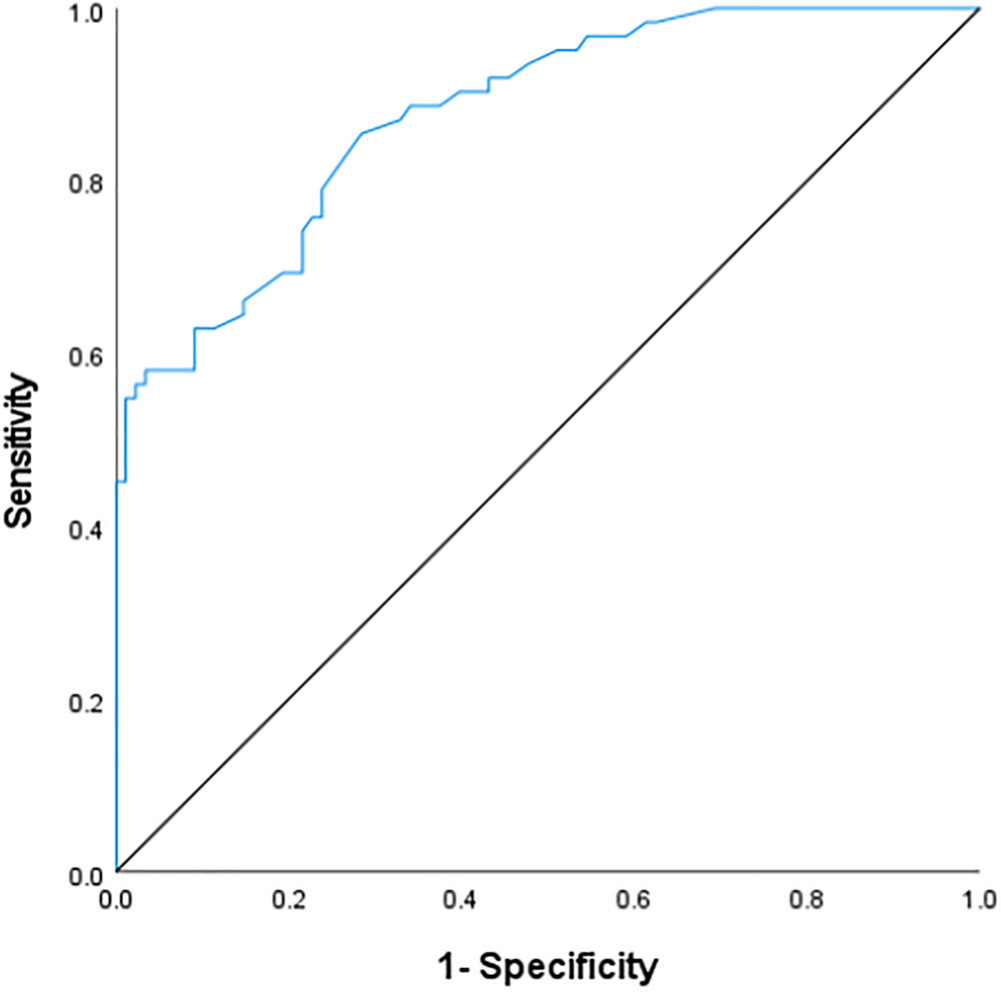
ROC curve of the predictive model.

The model was internally validated using the Bootstrap resampling method with 1000 iterations (*B* = 1000), and validation data are provided in Table 4. The predictive performance of the model was evaluated using the area under AUC, which yielded a value of 0.877 (95% CI: 0.823–0.931), indicating good discriminative ability (see Figure 2).

**TABLE 4:** Bootstrap resampling of variables in the predictive model equation.

Variables	*B*	Bias	S.E.	*P*	95% CI Lower limit	95% CI Upper limit
Vagus nerve injury (1)	0.944	0.061	0.517	0.035	0.045	2.062
Tracheotomy/cannulation (1)	1.143	0.050	0.568	0.025	0.064	2.292
Mechanical ventilation (1)	1.234	0.044	0.585	0.018	0.195	2.501
Aphasia severity	0.656	0.022	0.331	0.024	0.029	1.345
GCS score	−0.902	−0.066	0.162	0.001	−1.322	−0.698
Constant	5.103	0.450	1.544	0.001	2.969	9.006

**FIGURE 3:**
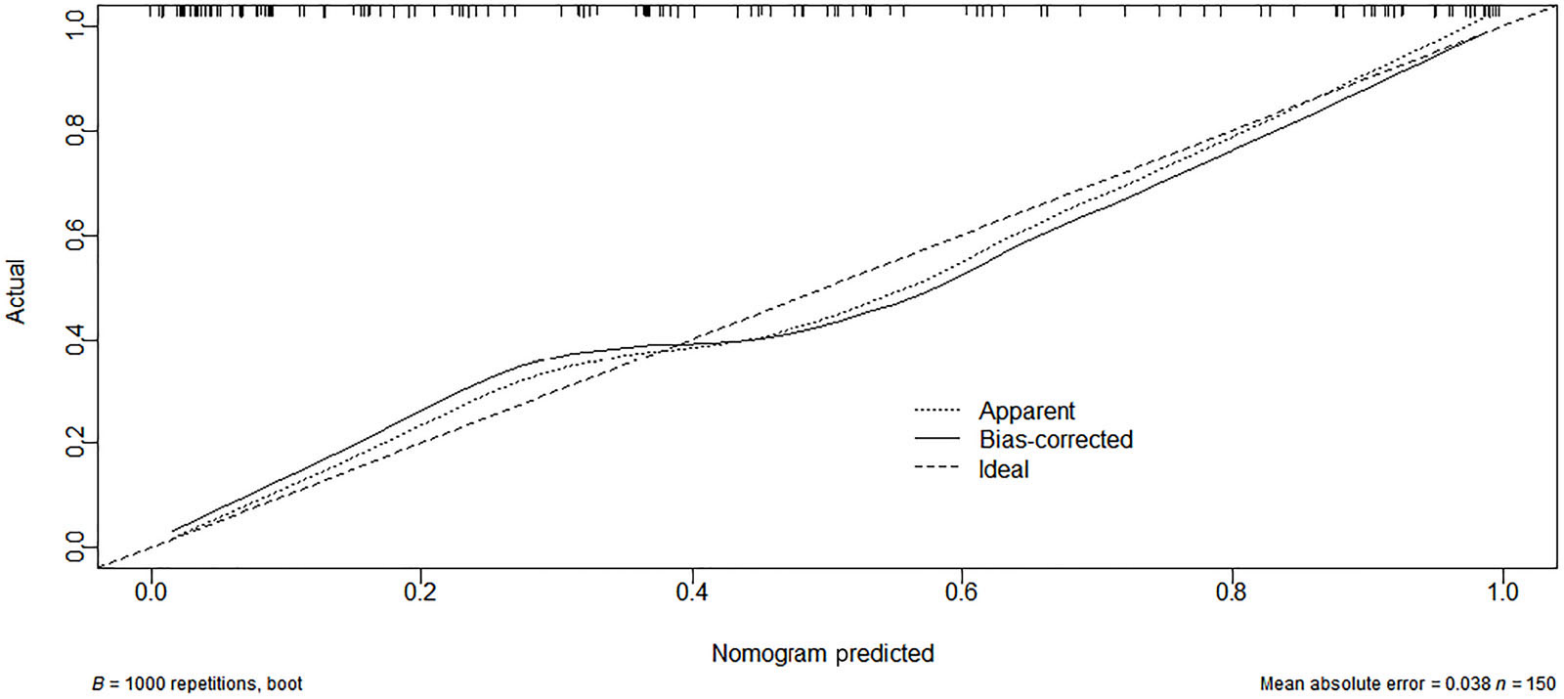
Calibration curve of the predictive model.

**FIGURE 4:**
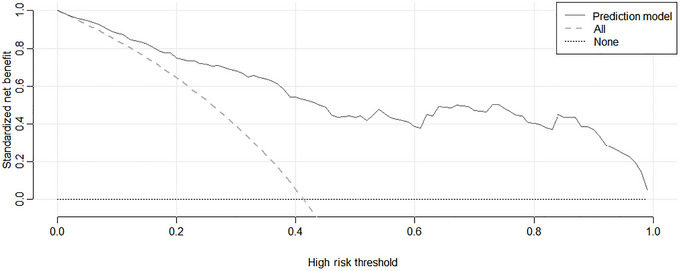
Decision curve analysis of the predictive model.

Decision curve analysis showed that the threshold probability range of the model was 1%–99%, with an absolute error of 0.038 between observed and predicted probabilities. There was no statistically significant difference between observed and predicted values (*X* ^2^ = 5.6728, *p* = 0.6838), confirming the model's good calibration and clinical applicability (see Figures 3 and 4).

## 4 Discussion

Craniocerebral injury refers to traumatic damage to the skull and brain parenchyma caused by direct or indirect external force to the head. According to statistics (Huang et al. 2024), craniocerebral injuries account for approximately 9%–21% of all body trauma cases, second only to limb fractures, but they rank first in terms of mortality and disability rates. Beyond damage to the scalp and skull, more critically, these injuries can lead to brain contusions, lacerations, hemorrhage, and other pathological changes that severely compromise neurological function and may be life‐threatening. Early intervention is therefore essential. Although treatment strategies have improved significantly over time, postoperative swallowing dysfunction has become increasingly prominent (Abbas et al. 2021). Dysphagia not only increases the risk of aspiration and aspiration pneumonia but also contributes to malnutrition, delays rehabilitation, reduces quality of life, and may induce fear of eating, social isolation, or depression, factors that can be fatal in severe cases. Thus, early detection and prevention are crucial (Mucuoglu et al. 2022).

Binary logistic regression analysis identified vagal nerve injury, tracheotomy/cannula, mechanical ventilation, and severe aphasia as independent risk factors for dysphagia in patients with craniocerebral injury, while GCS score emerged as a protective factor. The vagus nerve is essential for triggering the swallowing reflex. It innervates the pharyngeal and laryngeal muscles, including the soft palate, pharyngeal constrictors, and upper esophageal sphincter, coordinating muscle contraction and relaxation to enable safe passage of food or liquids into the esophagus (Tillmann et al. 2022; Kulesza et al. 2021). Vagus nerve injury may lead to muscle weakness or discoordination, impair neurotransmitter function, and hinder signal transmission, thereby reducing or eliminating the swallowing reflex and resulting in dysphagia (Tourigny et al. 2021). In univariate analysis, patients with dysphagia had significantly higher rates of tracheotomy, consistent with findings from Gallice et al. (2024) and Eskildsen et al. (2024). Tracheotomy disrupts the upper airway, allowing air to bypass the naso‐oropharynx and flow directly into the lower airway via the cannula, reducing mucosal sensitivity and impairing reflex initiation. Moreover, subglottic pressure is diminished post‐tracheotomy. Under normal conditions, positive subglottic pressure during swallowing helps clear residual food from the glottis and prevents aspiration. Loss of this pressure compromises this defense mechanism. Additionally, the presence of a tracheal cannula may restrict the upward movement of the larynx, a crucial component of the swallowing process that facilitates epiglottic closure and upper esophageal sphincter opening, thereby increasing the risk of dysphagia (Butterfield et al. 2023). Patients with dysphagia were also more likely to have undergone mechanical ventilation, consistent with Zuercher et al. (2020). Mechanical ventilation compromises the anatomical integrity of the upper airway and disrupts protective mechanisms during swallowing. Long‐term ventilated patients are often fed via nasogastric tubes to reduce aspiration risk, which leads to disuse atrophy of pharyngeal muscles and decreased swallowing strength. Cognitive dysfunction may impair pathways that trigger the swallowing reflex. Normally, sensory cues from vision and hearing initiate salivary and gastric secretions in preparation for eating. In patients with severe aphasia, cognitive impairment reduces the ability to interpret these cues and impairs cooperation. These patients may also have difficulty initiating voluntary oral and pharyngeal muscle movements due to corticobulbar tract damage or extrapyramidal system involvement, which increases local muscle tone and rigidity, both contributing to impaired bolus transit and dysphagia. GCS is a measure of consciousness. Higher GCS scores correlate with better pharyngeal muscle relaxation, airway closure, and reflex sensitivity, thus reducing aspiration risk. Lower GCS scores indicate more severe neurological impairment, which directly compromises the neural pathways involved in the swallowing reflex.

Based on these findings, targeted interventions are recommended. For instance, close monitoring of the patient's swallowing function is essential, particularly the functional status of areas innervated by the vagus nerve, such as the soft palate, pharyngeal constrictor muscles, and upper esophageal sphincter. Early signs of dysphagia should be promptly identified using assessment tools such as the Kubota WST. Swallowing function should be regularly evaluated to track recovery progress. Under physician supervision, neurotrophic agents such as B vitamins and mecobalamin may be administered to promote nerve repair and regeneration. Where feasible, electrical stimulation therapy should be employed to stimulate the vagus nerve and its branches, thereby enhancing neural conduction recovery. Meanwhile, personalized swallowing rehabilitation programs should be developed based on the degree of neural impairment. These may include sensory stimulation training (e.g., using foods of varying temperatures and textures to stimulate the oral and pharyngeal regions), cold‐hot oral brushing care (to enhance oral sensation and muscle control), and therapeutic touch (to improve local blood circulation and neural function). During nursing care, attention should be paid to proper positioning, especially during feeding. Patients should be assisted into a semi‐recumbent or seated position with a slight forward tilt of the head to facilitate pharyngeal passage of food and reduce the risk of aspiration. Food texture and feeding methods should be adjusted according to the patient's swallowing function recovery. Initially, pureed or semi‐liquid foods may be offered, gradually transitioning to soft and regular diets. For patients with severe dysphagia, timely implementation of enteral nutrition via feeding tube is necessary, with parenteral nutrition support as appropriate to ensure adequate hydration and nutritional intake. For tracheotomy/cannulation management, the patency of the tracheal cannula and regular clearing of airway secretions should be ensured to prevent aspiration and suffocation. During feeding, the patient's head or adopt specific body positions should be elevated to facilitate smooth transit of food through the pharynx. Oral care should be reinforced, including regular tooth brushing and mouth rinsing, to prevent oral infections. Additionally, regular assessments of respiratory and swallowing functions should be conducted. Mechanical ventilation parameters should be adjusted based on the patient's condition to minimize impact on swallowing function. When clinically permissible, early weaning from mechanical ventilation should be pursued to reduce interference with swallowing. After weaning, swallowing rehabilitation training should be promptly initiated to promote functional recovery. Moreover, communication aids such as pictures or gestures to help patients express their needs and emotions are needed. Cognitive training should also be conducted to improve attention and memory, which can indirectly support the recovery of swallowing function. Close monitoring of GCS scores is necessary to evaluate the patient's level of consciousness and neurological function. For patients with low GCS scores, oral feeding training and feeding opportunities should be increased as early as possible to stimulate swallowing recovery.

In summary, vagus nerve injury, tracheotomy/cannulation, mechanical ventilation, severe aphasia, and low GCS score are key risk factors contributing to dysphagia in patients with craniocerebral injury. Targeted interventions addressing these factors are essential for prevention and prognosis improvement. Although this study has established an effective predictive model, certain limitations remain. The relatively small sample size may affect the generalizability of the findings. Additionally, the long‐term efficacy of the proposed interventions was not tracked. Future studies should consider expanding the sample size and extending follow‐up duration to further validate and refine the model, thereby providing more robust clinical guidance. Several limitations of this study should be acknowledged. First, although multiple comorbidities (such as diabetes, chronic kidney disease, chronic pulmonary disease, and advanced cardiac dysfunction) and airway‐related factors (e.g., endotracheal tube size) have been associated with dysphagia in previous studies, these variables were not consistently available in our retrospective dataset and therefore could not be systematically incorporated into the primary risk factor model.

Second, some airway management–related parameters may function as intermediate variables rather than true confounders in the causal pathway linking neurological injury, respiratory support, and dysphagia. Adjusting for such post‐exposure or treatment‐related variables may introduce overadjustment or collider bias and potentially distort the estimated associations.

To mitigate confounding by overall illness severity, we performed extended analyses incorporating validated severity scores (APACHE‐II/SOFA) and duration of mechanical ventilation, which supported the robustness of the primary findings. Nevertheless, residual confounding due to unmeasured comorbidities cannot be entirely excluded. Future prospective multicenter studies with standardized collection of comorbid, airway, and severity‐related variables are needed to further refine risk stratification and optimize targeted nursing strategies. Importantly, many comorbidities and airway‐related factors may serve as markers of overall vulnerability rather than modifiable targets, whereas early identification of high‐risk patients based on neurological status and respiratory support characteristics may offer more practical value for targeted nursing interventions. The observed ventilation durations in our mechanically ventilated subgroup fall within the range reported in previous ICU studies and demonstrate a clear trend toward longer exposure among patients with dysphagia, further supporting the biological plausibility of the association. It should be noted that only a subset of patients in our cohort required invasive mechanical ventilation, which may limit the precision of effect estimates for ventilation duration. Larger prospective studies focusing on mechanically ventilated populations are warranted to further clarify dose–response relationships. Although WST is routinely implemented as part of standard ICU care, the retrospective nature of this study may still introduce residual selection bias, particularly in patients who were unable to undergo swallowing assessment due to early death or profound neurological impairment. Prospective studies with standardized assessment protocols are warranted to further address this limitation.

## Author Contributions

Q.Z. and Y.P. designed the research and drafted the manuscript. H.S. and F.Y. collected and organized data, contributed to the critical revision of the manuscript. All authors contributed to the manuscript and approved the submitted version.

## Funding

The authors have nothing to report.

## Ethics Statement

This study was approved by the ethics committee of Taizhou Affiliated Hospital of Nanjing University of Chinese Medicine (No. 20240711) and was conducted in accordance with the local legislation. The participants provided their written informed consent to participate in this study

## Conflicts of Interest

The authors declare no conflicts of interest.

## Supporting information




**Supporting Information**: brb371347‐sup‐0001‐TableS1.docx

## Data Availability

The data that support the findings of this study are available from the corresponding author upon reasonable request.
