# Supplementary material for: Risk Factor Analysis and Prognosis of Dysphagia in Craniocerebral Injury Patients: Implications for Targeted Nursing Strategies
Source: Brain Behav. 2026 Mar 26;16(4):e71347. doi: 10.1002/brb3.71347 (PMC13112032; doi:10.1002/brb3.71347)
Supplement: Supplementary file 1 — Supporting Information: brb371347‐sup‐0001‐TableS1.docx [file BRB3-16-e71347-s001.docx]

**Supplementary Table 1.** Sensitivity and stratified analyses examining the robustness of the association between mechanical ventilation, baseline GCS, and dysphagia

| **Baseline GCS category** | **n** | **OR for mechanical ventilation** | **95% CI** | **P value** |
| --- | --- | --- | --- | --- |
| ≤ 7 | 34 | 4.777 | 0.215–106.227 | 0.323 |
| 8–9 | 62 | 2.613 | 0.848–8.056 | 0.094 |
| ≥ 10 | 54 | 2.867 | 0.349–23.562 | 0.327 |

Sensitivity analysis was performed by excluding patients with extremely low baseline neurological status (baseline GCS ≤ 6) and repeating the multivariable logistic regression model. Stratified analyses were conducted according to baseline GCS categories (≤7, 8–9, and ≥10). Odds ratios (ORs) greater than 1 indicate an increased risk of dysphagia associated with mechanical ventilation. The attenuation of statistical significance in stratified analyses is likely due to reduced sample sizes within subgroups.
